# Osthole: A Coumarin Derivative Assuage Thiram-Induced Tibial Dyschondroplasia by Regulating *BMP-2* and *RUNX-2* Expressions in Chickens

**DOI:** 10.3390/antiox8090330

**Published:** 2019-08-22

**Authors:** Muhammad Waqas, Yaping Wang, Aoyun Li, Hammad Qamar, Wangyuan Yao, Xiaole Tong, Jialu Zhang, Mudassar Iqbal, Khalid Mehmood, Jiakui Li

**Affiliations:** 1 College of Veterinary Medicine, Huazhong Agricultural University, Wuhan 430070, China; 2 Faculty of Veterinary & Animal Sciences, University of the Poonch, Rawalakot, District Poonch 12350, Azad Jammu & Kashmir, Pakistan; 3 University College of Veterinary & Animal Sciences, Islamia University of Bahawalpur, Bahawalpur 63100, Pakistan; 4 College of Animal Husbandry and Veterinary Medicine, Tibet Agricultural and Animal Husbandry University, Linzhi 860000, China

**Keywords:** biochemical markers, genes, growth plate, liver antioxidants, osthole, tibial dyschondroplasia

## Abstract

Avian tibial dyschondroplasia affects fast growing broiler chickens accounting for almost 30% of leg ailments in broilers. The present project was designed to assess the efficacy of osthole against avian tibial dyschondroplasia (TD). Two hundred and forty chickens were equally allocated into control, TD and osthole groups (n = 80). The TD and osthole group chickens were challenged with tetramethylthiuram disulfide (thiram) at 50 mg/kg of feed from 4–7 days, followed by osthole administration at 20 mg/kg orally to the osthole group only from 8–18 days. Thiram feeding resulted in lameness, increased mortality, and decreased production parameters, alkaline phosphatase (ALP), superoxide dismutase (SOD), total antioxidant capacity (T-AOC), and glutathione peroxidase (GSH-PX) levels, along with significantly increased aspartate aminotransferase (AST), alanine aminotransferase (ALT), malondialdehyde (MDA) levels, and growth plate size. Moreover, the genes and protein expressions of *BMP-2* and *RUNX-2* were significantly down-regulated in TD affected chickens (*p* < 0.05). Osthole administration showed promising results by alleviating lameness; increased ALP, SOD, T-AOC, and GSH-Px levels; and decreased the AST, ALT, and MDA levels significantly. It restored the size of the growth plate and significantly up-regulated the *BMP-2* and *RUNX-2* expressions (*p* < 0.05). In conclusion, the oxidative stress and growth plate anomalies could be assuaged using osthole.

## 1. Introduction

The poultry industry is extremely important for creating a sustainable livelihood and economic independence. It provides animal proteins, i.e., chicken, meat, and eggs, that are widely consumed by the human population [1]. The leg ailments are considered the biggest economic threats to the poultry industry and many of these abnormalities are associated with interference in the maturational sequence of chondrocytes within the growth plate [2]. Avian tibial dyschondroplasia is one of these abnormalities [2] accounting for almost 30% of leg ailments in broilers [3]. It results in abnormal differentiation of growth plate (GP) chondrocytes responsible for cartilage vascularization, mineralization, and bone formation [4]. The definite cause of TD is still obscure but factors causing angiogenesis retardation have been recognized to induce its occurrence [5]. Thiram is an eminent pesticide, fungicide, and fly and rodent repellent [4,6]. Owing to its diffusion in the soil, atmosphere, and water [7], the pervasive usage of such agents is life-threatening [8]. Its inclusion into the poultry food chain is a potential menace to the poultry industry [9] and can cause feed toxicity that in turn leads the chickens to develop tibial dyschondroplasia. [6,10]. It has harmful and noxious effects on the liver [11] and halts angiogenesis [6]. Moreover, thiram feeding can experimentally induce tibial dyschondroplasia in chickens [12].

*BMPs* (bone morphogenetic proteins) are members of the transforming growth factor beta (TGF-β) superfamily [13]. These proteins are responsible for bone regeneration and differentiation [14] and have a key role in bone care and restoration [15]. *BMPs* expression elicits osteogenic signals driving the bone differentiation process [16]. Among *BMPs, BMP-2* is the earliest detected *BMP* having a stout in vivo and in vitro osteoinductive competence [17,18]. It is an eminent stimulator of bone development and osteoblasts differentiation [19]. Similarly, *RUNX-2* (runt-related transcription factor-2) is a vital transcription factor for chondrocyte maturation [20]. It is an obligatory gene for osteoblast differentiation [21] and a key bone regulator [22].

**Figure 1:**
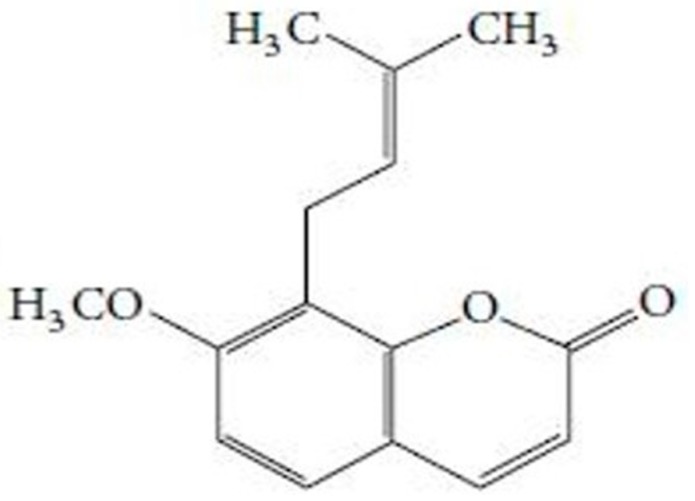
Chemical structure of osthole, [7-methoxy-8-(3-methyl-2-butenyl)-2H-1-benzopyran-2-one], the principle component of *Cnidium monnieri (Fructus cnidii)* [31].

Traditional Chinese medicines (TCMs) are used either individually or in combination against many diseases [23]. These medicines have been used effectively in the treatment of metabolic disorders [24]. Osthole is a coumarin derivative [25]. It is commonly called “She-Chuang-Zi,” which is derived from the dried fruit of *Cnidium monnieri (Fructus cnidii)* [26]. It has anti-inflammatory, anti-osteoporotic, anti-tumor [27], anti-oxidant, and anti-apoptotic properties [28], and is used in the management of allergies and asthma [29], inflammation, and vascular diseases [30]. In view of such eminent therapeutic potential, we hypothesized that osthole may prove to be an excellent alternative to synthetic drugs for the treatment of different ailments with special reference to tibial dyschondroplasia in chickens. Therefore, the current project was designed to check the in vivo therapeutic effects of osthole on survival rate, oxidative stress, liver toxicity, production performances, growth plate anomalies, and the genes and protein expression of *BMP-2* and *RUNX-2* in broiler chickens affected with tibial dyschondroplasia. The chemical structure of osthole is shown in Figure 1 [31].

## 2. Materials and Methods

### 2.1. Animal Ethics

The animal trial was conducted under the approval of the ethics committee of the Huazhong Agricultural University Wuhan, P.R China, by strictly taking into consideration all the national legislation and protection of animal welfare concerns (approval No. 31273519).

### 2.2. Experimental Birds, Chemical Reagents, and Medicine

Two hundred and forty broiler chicks (average weight 41 ± 2 g) were purchased from a hatchery (Chia Tai Animal Husbandry Co. Ltd., Jingzhou, China). Thiram was purchased from Shanghai Macklin Biochemical Co. Ltd., Shanghai, China. Commercial reagents kits for superoxide dismutase (SOD), glutathione peroxidase (GSH-Px), total antioxidant capacity (T-AOC), and malondialdehyde were purchased from Nanjing Institute of Biological Engineering, Inc., Jiangsu, China. Reagents kits for aspartate aminotransferase (AST), alanine aminotransferase (ALT), and alkaline phosphatase (ALP) were purchased from Biosino Biotechnology and Science Inc., Beijing, China. Fluid for bone demineralization (Cat: B1023) was purchased from Powerful Biology, Wuhan, China. Osthole (Lot: T01M9B54821, purity ≥ 98%) was purchased from Shanghai Yuanye Biotechnology Co. Ltd., Shanghai, China. Trizol reagent was procured from Invitrogen, Carlsbad, CA, USA. The First-Strand cDNA synthesis kit was procured from TransGen Biotech, Beijing, China. Primers for *BMP-2* and *RUNX-2* genes and the reference gene glyceraldehyde 3-phosphate dehydrogenase *(GAPDH)* were synthesized by GenScript^®^, Nanjing, China. The bicinchoninic acid assay (BCA) protein detection kit and rabbit polyclonal *anti-BMP-2* (servicebio GB: 11252) antibodies were purchased from Service Biotechnology, Wuhan, China. The rabbit polyclonal *anti-RUNX-2* (Abcam: ab23981) antibodies were purchased from Abcam Trading Company Ltd., Shanghai, China.

### 2.3. Experiment Design

**Figure 2:**
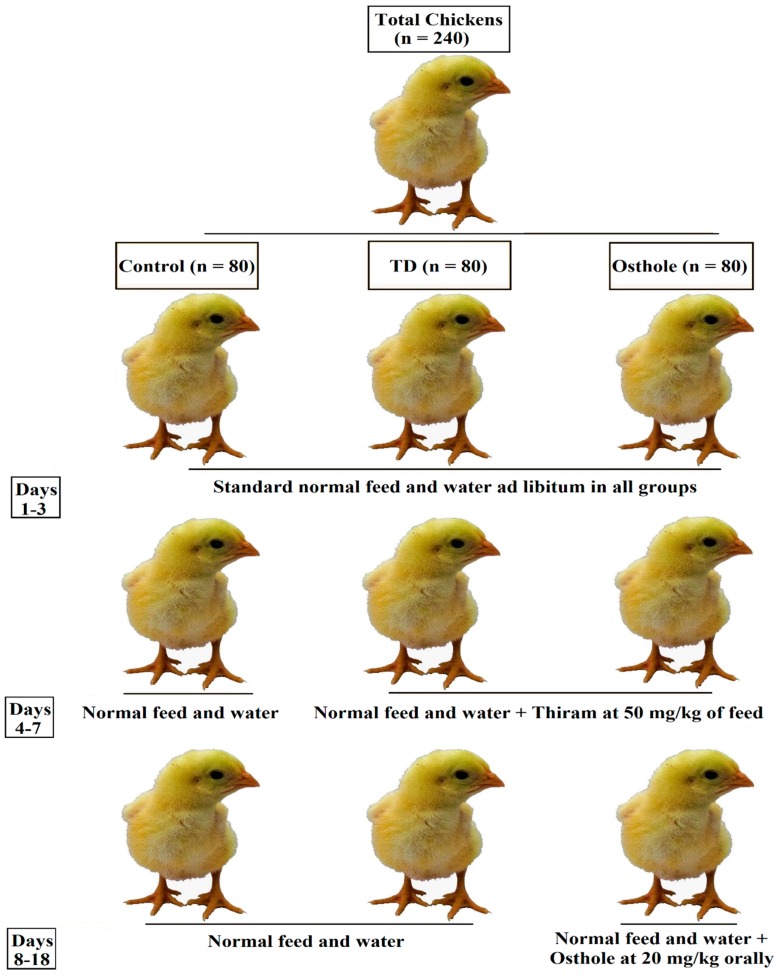
Experiment design.

The schematic experiment design is explained in Figure 2 [32]. An equal number of chickens were incorporated into three groups designated as control, TD, and osthole groups (n = 80). The total duration of the experiment was 18 days. Throughout the experiment, control group chickens received standard normal feed and water. However, the TD and osthole groups’ chickens were challenged with thiram at 50 mg/kg of feed from days 4–7 [9,33]. On day 8, the thiram feeding was discontinued in both the TD and osthole groups followed by treating osthole group chickens only with osthole at 20 mg/kg orally from days 8–18 [27,34].

### 2.4. Sample Collection

Samples were collected on the 7th, 10th, 14th, and 18th days. Fifteen birds (n = 15) were randomly selected from each group on each specified day. Before sacrificing, blood samples were collected via jugular venipuncture, which were then centrifuged at 3000× *g* for 20 min for serum separation and stored at −70 °C for later analysis of biochemical parameters. Afterward, the chickens were sacrificed using cervical dislocation and were then dissected to collect liver, kidney, spleen, heart and bone samples. The liver, spleen, kidney, heart, and bone samples were stored immediately at −80 °C. The liver was used for the assessment of the oxidative stress and bones for the genes and protein expression and immunohistochemistry. Some of the tibiotarsal bone samples were fixed in 4% paraformaldehyde for later use in hematoxylin and eosin staining. The visceral organs, i.e., liver, spleen, kidney, and heart were used for the measurement of visceral organs indices.

### 2.5. Mortality Assay, Production Parameters, and Visceral Organs Indices

The mortality was noted daily and the parameters regarding average daily weight gain, average daily feed intake, and feed conversion ratio (FCR) were recorded on various days. The liver, kidney, spleen, and cardiac indices were measured in all the groups on various days. The visceral organs indices were calculated as their weight per body weight of chicken [6].

### 2.6. Biochemical and Antioxidants Analyses

The activity of ALP and the levels of AST and ALT in the serum samples were assessed in the control, TD, and osthole groups via commercial kits using a semiautomatic biochemical analyzer (Coulterr LH 750, Guangdong, China) as per the instruction manuals and the values were presented in units per liter (U/L) as per previous studies [4]. Meanwhile, SOD, T-AOC, and GSH-PX activity, as well as MDA contents, were assessed in the liver samples of all the groups. Concisely, a liver homogenate was prepared with the help of a polytron aggregate homogenizer (Polytron PT-MR 3100, KINEMATICA AG, Luzern, Switzerland) by adding a 10 mM phosphate buffered saline (10 mL PBS/0.1 g of tissue). The homogenate was then centrifuged at 3500× *g* for 10 minutes and the supernatant was collected for the determination of liver antioxidants with UV spectrophotometer using assay kits according to instruction manuals. The values for liver SOD, T-AOC, and GSH-Px were expressed in U/mg (U per milligram of protein), while liver MDA contents were interpreted in nmol/mg (nanomoles per milligram) [35,36].

### 2.7. Tibia Bone Parameters

Tibia parameters, i.e., the length and weight of tibia, growth plate width, and tibia index were recorded in control, TD, and osthole groups. A ruler, an electronic balance and digital calipers (#SATA91511, TATA Company, Shanghai, China) were used to measure the length, weight, and GP width respectively [6]. The tibia index was calculated as the tibia weight divided by chicken weight before slaughtering [37].

### 2.8. Hematoxylin and Eosin (H&E) Staining and Immunohistochemistry

The tibiotarsal bone samples fixed in 4% paraformaldehyde were then decalcified in bone demineralization fluid, dehydrated in ethanol, cleared in xylene, and embedded in paraffin wax. Histological slides were prepared by cutting the growth plate sections into 5-μm thick slices, followed by dewaxing in xylene and staining with hematoxylin and eosin stain for histological analysis [10]. Immunohistochemical analysis was performed according to an earlier described method [38]. After washing with PBS and peroxidase blocking solution (Boster, Wuhan, China), the slides were incubated with *anti-BMP-2* and *anti-RUNX-2* primary antibodies (1:1000) overnight at 4 °C. After washing with PBS, they were again incubated with secondary antibodies (1:200), this time in the dark for 2 hours at 25 °C. Finally, the slides were then examined under the microscope (Olympus CX31, Tokyo, Japan).

### 2.9. Reverse Transcription Quantitative Polymerase Chain Reaction (RT-qPCR)

The RNA extraction and RT-qPCR were conducted as per previous studies [39]. The total RNA from GP tissues was extracted using the Trizol method. After calculating the concentration of extracted RNA with a Nanodrop 2000 analyzer (Thermo scientific, Waltham, MA, USA), the total RNA with a final volume of 20 μL was then reversely transcribed to cDNA using a cDNA synthesis kit. The RT-qPCR reactions were performed in quadruplex with a Step One-Plus™ qRT-PCR system (Applied Biosystems, Foster City, CA, USA). The sequences of the primers used in this study are given in Table 1. The relative quantification of gene expression was measured using the delta Ct (2^−ΔΔCt^) method [40].

### 2.10. Western Blotting

Western blotting was performed as per previous studies [37,39]. Briefly, after the homogenization of growth plates in ice-cold PBS and storage at 4°C for 2 hours, the supernatant was collected after centrifugation at 14,000× *g* for 10 min. Total protein concentration was determined using a BCA kit and the samples were stored at –70 °C. The equal amount of proteins from growth plates were separated using 10% sodium dodecyl sulfate–polyacrylamide gel electrophoresis (SDS-PAGE) and then transferred to polyvinylidene difluoride (PVDF) membranes. The membranes were incubated in 5% skimmed milk for 1.5 hours at room temperature and then incubated at 4 °C overnight with *BMP-2* and *RUNX-2* primary antibodies (1:1000). The membranes were then washed with tris-buffred saline tween (TBST) for 5 min and incubated with secondary antibodies (1:3000) at room temperature for 30 min, followed by washing with TBST four times. By using the β-actin as a loading control, finally, the images were taken with an imaging system (Ultra-Violet Products Ltd., Upland, CA, USA).

### 2.11. Statistical Analyses

The data was analyzed using one-way analysis of variance (ANOVA) and Student’s *t*-test using SPSS Statistical Package (v19.0, SPSS Inc., Chicago, IL, USA). All the figures were created using Graphpad Prism 6 (GraphPad Software Inc., San Diego, CA, USA). The data were expressed as the mean ± standard deviation (mean ± SD). The differences were considered statistically significant if *p* < 0.05.

## 3. Results

### 3.1. Clinical Observations of Thiram–Induced TD

**Figure 3:**
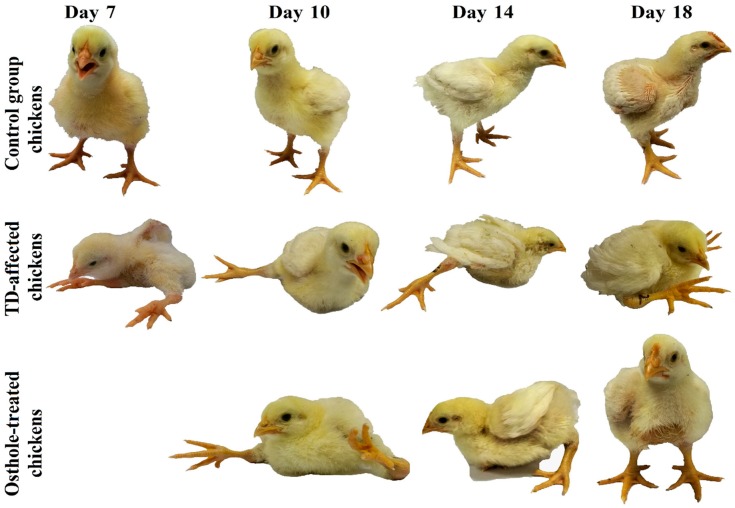
Birds showing characteristic clinical signs of lameness in the TD and osthole groups after thiram feeding compared to the control group. Osthole provided efficient recovery in osthole group chickens.

All the chickens were strictly monitored throughout the trial period for any obvious clinical signs. Up to three days of consuming the normal standard feed, there were no signs of any deformities among chickens in any of the groups. However, when the chickens in the TD and osthole groups were fed with thiram from the 4th to 7th day, the chickens developed a variety of clinical signs like depression, leg deformities, lameness, and difficulty in standing. In contrast, the control group chickens were physically active and looking healthy. After thiram stoppage, the clinical signs in the TD group reduced in severity but persisted throughout the experimental period. On the other hand, the osthole group chickens were able to eat, drink, stand on their feet, and walk properly after treatment with osthole (Figure 3).

### 3.2. Chicken Mortality and Survival Rate

**Figure 4:**
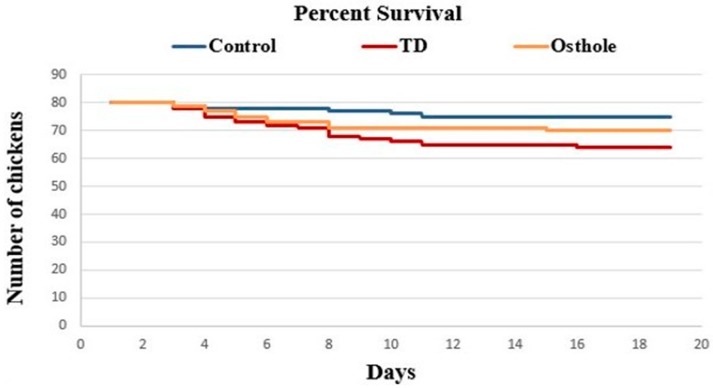
Effect of osthole on the survival rate of chickens. The osthole group chickens that were initially challenged with thiram from days 4–7 were treated with osthole from days 8 to 18.

The mortality among groups was recorded on daily basis, i.e., from 1–18 days (Table 2). It was evident that there was a drastic increase in the number of dead birds in the TD group. The mortality rates in the control, TD, and osthole groups were 6.25% (5/80), 20% (16/80), and 12.5% (12/80) respectively. This increased mortality rate was attributed to thiram administration in the TD and osthole groups. However, fewer dead birds were recorded in the osthole group, chiefly after the osthole administration (Figure 4).

### 3.3. Production Parameters Analysis

**Figure 5:**
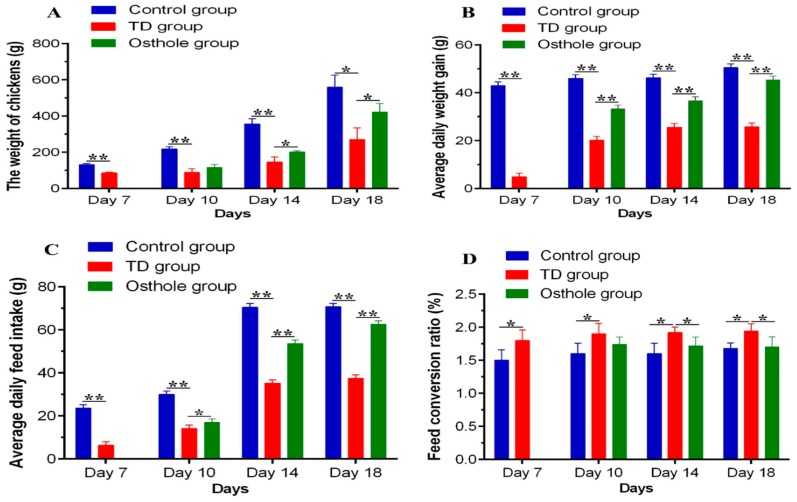
Production parameters analysis among groups on the 7th, 10th, 14th, and 18th days: (**A**) AW, (**B**) ADWG, (**C**) ADFI, and (**D**) FCR. The data are expressed as the mean ± SD. * *p* < 0.05. The standard FCR value in this study was 1.6.

The daily weight of chickens (DW), average daily weight gain (ADWG), average daily feed intake (ADFI), and the feed conversion ratio (FCR) were recorded. A significant decrease in the growth parameters of TD affected chickens was observed on various days (*p* < 0.05). However, after the osthole administration, these growth indices were significantly improved in the osthole group chickens (*p* < 0.05). Moreover, the FCR of the TD group chickens was poor, signifying a poor weight gain and feed intake. Conversely, the FCR of the osthole group was poor on day 10 but gradually improved on days 14 and 18 after the osthole administration (*p* < 0.05) (Figure 5).

### 3.4. Tibia Parameters Analysis

**Figure 6:**
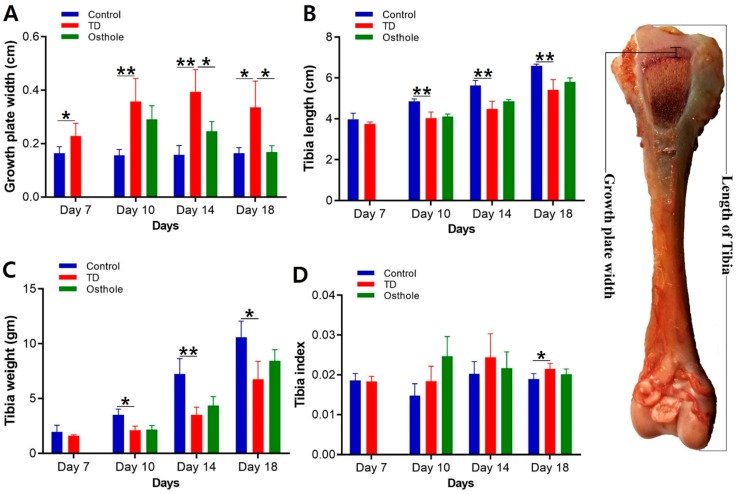
Tibia parameter analysis of the control, thiram, and osthole groups on various days, * *p* < 0.05: (**A**) growth plate width, (**B**) tibia length, (**C**) tibia weight, and (**D**) tibia index.

It was evident that there was significantly decreased length and weight of the tibia bone and increased width of the growth plate in TD affected chickens along with an increased tibia index (*p* < 0.05). Osthole significantly restored the width of the growth plate (*p* < 0.05). The length and weight of the tibia and the tibia index in the osthole group were nearly normal, i.e., similar to the control group, but the results were non-significant (Figure 6).

### 3.5. Serum Biochemical and Liver Antioxidants Analyses

**Figure 7:**
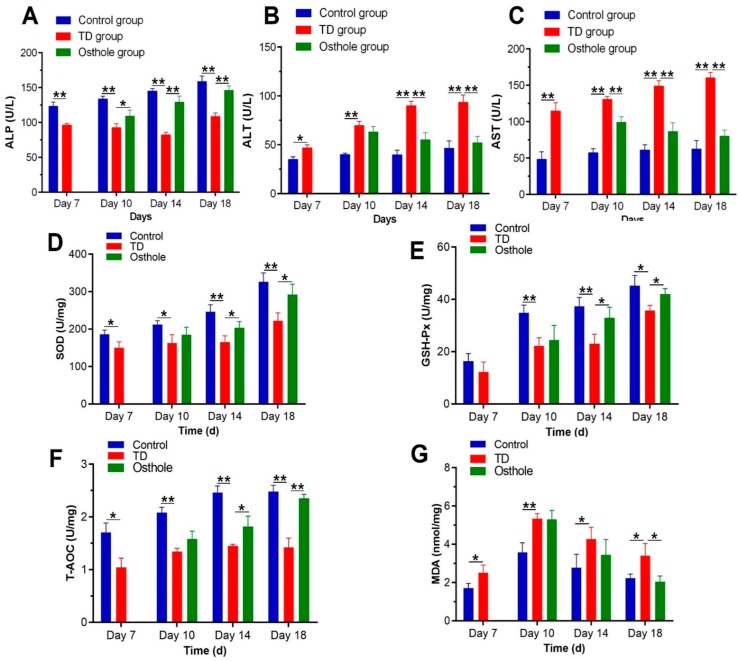
Effect of thiram on serum biochemical and liver antioxidant parameters followed by excellent recovery by osthole in the control, TD, and osthole groups: (**A**) ALP, (**B**) ALT, (**C**) AST, (**D**) SOD, (**E**) GSH-Px, (**F**) T-AOC, and (**G**) MDA. There was a notable difference in values for different days. The data are expressed as the mean ± SD (* *p* < 0.05).

After thiram administration, the ALP activity was significantly decreased along with elevated AST and ALT levels in thiram and osthole group chickens as compared to the control group (*p* < 0.05). Moreover, there was a prominent oxidative imbalance in TD-affected chickens in terms of decreased SOD, GSH-Px, and T-AOC levels, along with increased MDA contents. Osthole administration significantly normalized the ALP activity and ALT and AST levels, and relieved the oxidative stress in osthole group chickens, chiefly on the 18th day (*p* < 0.05) (Figure 7).

### 3.6. Visceral Organs Indices

**Figure 8:**
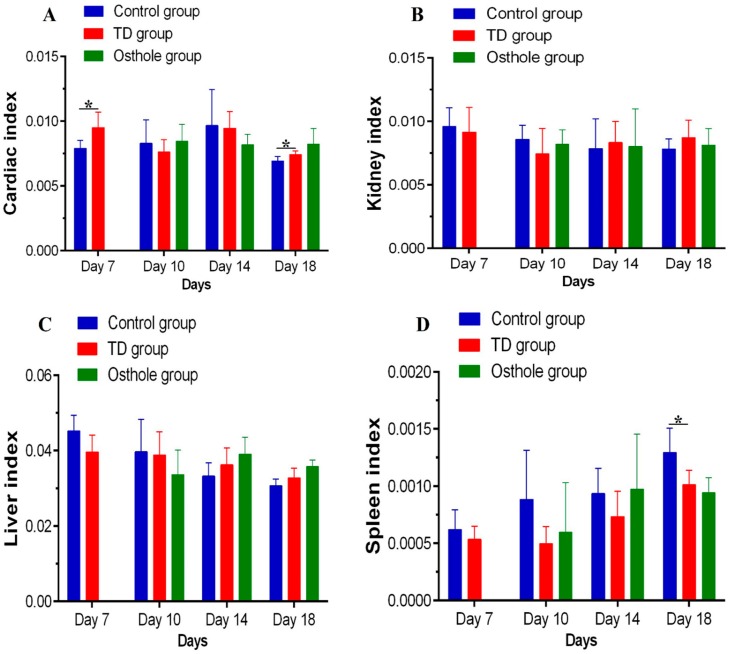
Visceral organs indices among groups on various days: (**A**) cardiac index, (**B**) kidney index, (**C**) liver index, and (**D**) spleen index.

The liver, spleen, heart, and kidney indices were calculated on the 7th, 10th, 14th, and 18th days. Overall, no significant difference was observed in these parameters among the groups. Nevertheless, there was a significant difference in the cardiac index on the 7th and 18th days and in the spleen index on the 18th day between the control and TD groups (*p* < 0.05) (Figure 8).

### 3.7. Histological Examination of the Tibial Growth Plates

**Figure 9:**
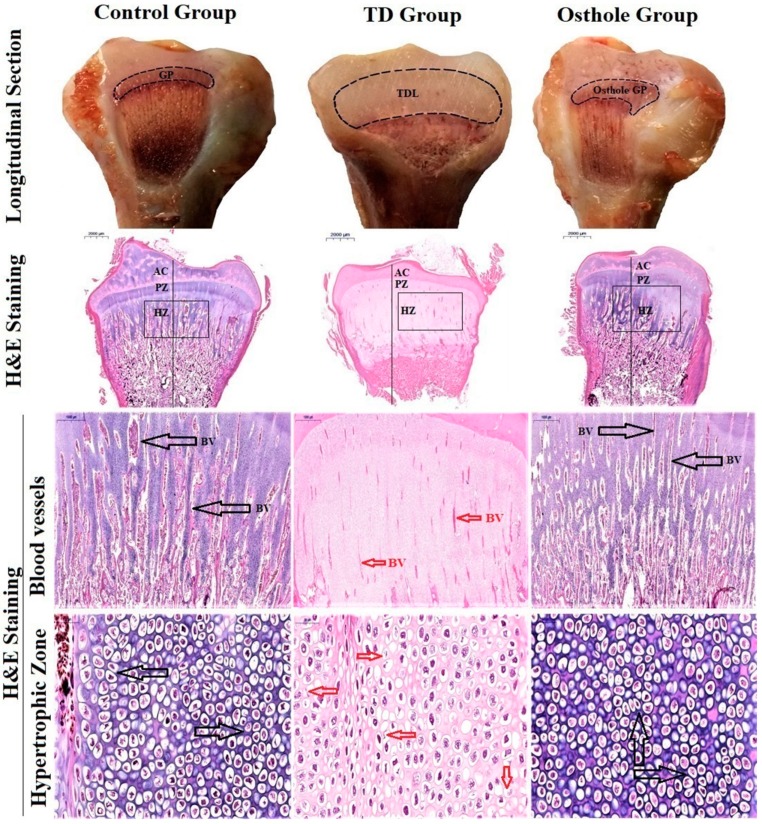
Growth plate morphological changes in the tibia (longitudinal section). H&E staining of tibia bones. The black arrows indicate blood vessels and a firmly arranged column of chondrocytes in the control and osthole groups. The red arrows indicate wrecked and dead cells having a disintegrated nucleus with less vascularity in the TD group. Key: Growth plate (GP), blood vessels (BV), tibial dyschondroplasia lesion (TDL), articular cartilage (AC); proliferative Zone (PZ), hypertrophic zone (HZ).

Hematoxylin-and-eosin-stained histopathological micrographs indicated enormous vascularity in proliferative and hypertrophic zones with normal tibia GP in the control group along with regular and tightly arranged chondrocytes with the nucleus at the center. Conversely, after thiram feeding, the vascularity in the TD growth plate was lost and the chondrocytes were distorted and necrotized. However, osthole administration from the 8–18th days efficiently restored the tibia GP and led to a proper columnar arrangement of the chondrocytes with huge progressing blood vessels in the hypertrophic zone of the osthole group (Figure 9).

### 3.8. Immunohistochemical Analysis

**Figure 10:**
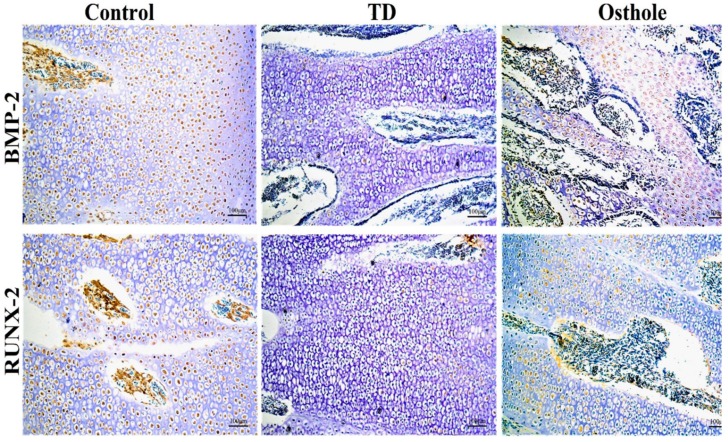
Immunohistochemical localization of *BMP-2* and *RUNX-2* antibodies in the control, TD, and osthole groups.

The localizations of *BMP-2* and *RUNX-2* antibodies in the growth plates of control, TD, and osthole groups were checked using immunohistochemistry. There were a greater number of cells positively stained with *BMP-2* and *RUNX-2* antibodies in the control and osthole-treated groups. Conversely, the TD group had fewer positively stained cells (Figure 10).

### 3.9. The mRNA and Protein Expressions of BMP-2 and RUNX-2

**Figure 11:**
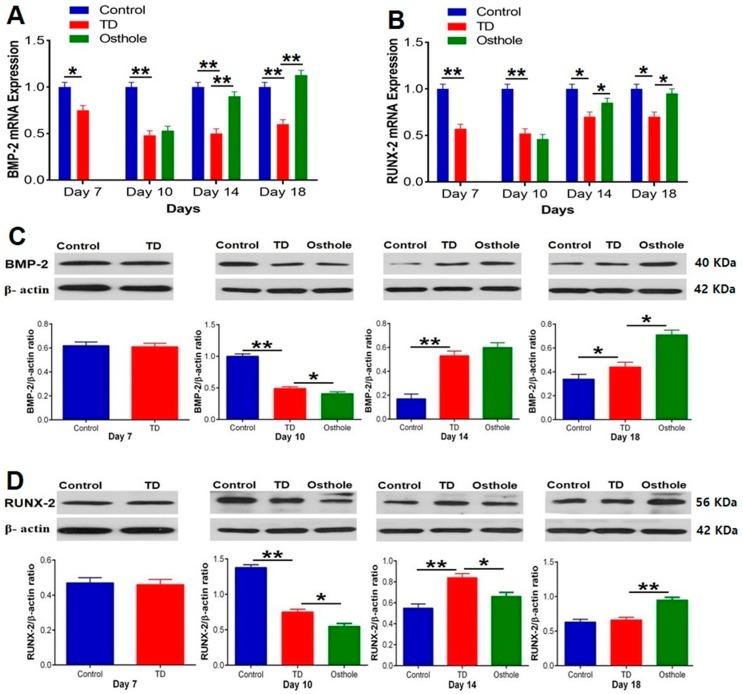
RT-qPCR and western blot analysis of *BMP-2* and *RUNX-2* in the control, thiram, and osthole groups: (**A**) *BMP-2* mRNA expression, (**B**) *RUNX-2* mRNA expression, (**C**) *BMP-2* protein expression, and (**D**) *RUNX-2* protein expression. The bands were quantified using Image Studio Lite 5.2.5^®^ (LI-COR Biosciences, Lincoln, NE, USA). The data are expressed as the mean ± SD (* *p* < 0.05). TD—Tibial dyschondroplasia.

The mRNA expressions of the targeted genes and protein levels were scrutinized by conducting RT-qPCR and western blotting techniques. Significantly abridged mRNA expressions of *BMP-2* and *RUNX-2* were observed in the TD affected chickens (*p* < 0.05). Osthole administration gradually improved the mRNA expression and protein levels of *BMP-2* and *RUNX-2* with significant up-regulation on days 14 and 18 in the osthole group. The western blotting results followed almost the same pattern. The protein levels of *BMP-2* and *RUNX-2* were significantly down-regulated in the TD group, followed by significant up-regulation in the osthole group on various days (*p* < 0.05) (Figure 11).

## 4. Discussion

Thiram toxicity results in various distinct clinical manifestations in affected chickens, i.e., decreased physical activities [41], swollen tibia and death [18], and a reduced length and weight of the tibia bone with increased GP size and tibia index compared to normal healthy chickens [37,38]. Moreover, it has been reported that thiram can induce tibial dyschondroplasia (TD) [37] with a resultant reduced weight gain and feed intake and poor feed conversion ratio (FCR) [35,38]. In our study, compared to the control group, TD-affected chickens showed clinical signs like depression, leg distortions, lameness, standing difficulties, and increased mortality. The tibia length and weight were reduced and the growth plate width and tibia index were markedly increased. Chicken production parameters, i.e., daily weight, average daily weight gain, and average daily feed intake were decreased, along with having a poor feed conversion ratio. The alteration in production parameters was attributed to the fact that thiram caused lameness and stress, which in turn led the chickens to be deprived of feeding. Osthole relieved these clinical manifestations, reduced the mortality, restored the tibia parameters, and improved chicken performance effectively.

Variations in the levels of serum biochemical markers/enzymes, i.e., ALP, AST, and ALT typically reflects the health of liver. Lowered levels of ALP are reported to occur in osteoporosis [42]. Liver damage causes an increased release of ALT and AST into the blood circulation by damaged hepatocytes, causing their levels to rise [43,44]. In the current study, the levels of serum enzymes were assessed to evaluate the liver damage due to thiram toxicity. There were significant variations in the ALP, AST, and ALT levels in TD-affected chickens revealing a possible liver injury. These findings are in accordance with previous studies where a decreased ALP and increased ALT and AST levels were reported in thiram-induced TD chickens [12,45]. After the osthole treatment, the levels of serum enzymes were significantly restored. Our findings are in accordance with previous studies where osthole has been documented to augment the ALP activities [16] and is capable of reducing the higher levels of ALT and AST in rat models with hepatic injuries [46,47].

Both aerobic metabolism and pathological disorders result in the production of oxidants [48]. In a normal health status, there is always a balance between oxidants and antioxidants [49]. Any sort of disparity between these two is referred to as oxidative stress [48]. The liver is an important organ contributing almost 2% of the total body weight of an animal [50]. Having a key role in the detoxification of toxic compounds, the liver is vulnerable to many metabolic disorders for which the oxidative stress is the foremost clinical indicator [51]. The antioxidant enzymes, i.e., superoxide dismutase (SOD), provides an imperative antioxidant protection [52] by partitioning the superoxide anion free radical (O_2_^−^), thus decreasing the O_2_^−^ level responsible for the cell damage [53]. GSH-Px, having peroxidase activity, reduces the lipid hydroperoxides to alcohols and free hydrogen peroxide to water [54]. Total antioxidant capacity (TAC) is also used for assessing the antioxidant status and response against the radicals produced in the diseased condition [55]. Thiram causes oxidative stress and decreases the levels of these enzymes [12,56,57]. Oxidative stress affects the lipid peroxidation of the cell membrane with resultant overproduction of malondialdehyde (MDA) [58]. MDA is an extremely noxious end-product [59] and its level is an oxidative stress indicator [60]. Our results showed a significant decrease in SOD, GSH-Px, and T-AOC contents, along with a marked increase in MDA contents, indicating oxidative stress. Osthole treatment resulted in a marked assuagement of oxidative stress by bringing the abnormal oxidant levels to nearly normal, thus confirming that osthole has promising antioxidant effects [27,28,34,61].

Tibial dyschondroplasia is a bone anomaly in broiler chickens [62]. Hematoxylin and eosin staining revealed that TD resulted in less vascular cartilage with a noticeable number of cells having a pycnotic nucleus in proliferative and hypertrophic zones compared to the growth plates of control group chickens. Immunohistochemistry indicated fewer cells positively stained with *BMP-2* and *RUNX-2* antibodies in the TD group. Moreover, the expressions of *BMP-2* and *RUNX-2* were significantly down-regulated in TD-affected chickens compared to the control group. Our results are comparable to the previous studies where it has been reported that thiram results in the down-regulation of *BMP-2* and *RUNX-2* [18,39]. Osthole administration gradually improved the columnar alignment of cells in the growth plates of osthole group chickens along with an excessive number of cells positively stained with *BMP-2* and *RUNX-2* antibodies. Moreover, osthole administration significantly up-regulated the *BMP-2* and *RUNX-2* expressions in the osthole group chickens. Our findings are similar to previously reported studies where osthole has been reported to promote osteogenesis by up-regulating the expression of *BMP-2* [16,63,64] and *RUNX-2* [13,16,65].

## 5. Conclusions

In conclusion, osthole effectively averted the lameness and oxidative stress and enhanced the productions performance of chicken affected with tibial dyschondroplasia. Furthermore, it regulates the *BMP-2* and *RUNX-2* expressions, which are important for bone regeneration and osteoblast differentiation. Keeping in view the economic losses associated with tibial dyschondroplasia, osthole provides new insights toward the therapeutic options in the prophylaxis and treatment of bone maladies, particularly tibial dyschondroplasia in chickens. Altogether, it is an excellent traditional Chinese medicine possessing substantial therapeutic properties and can be used as an alternative medicine to synthetic drugs that are costly and have enormous side effects.

## Figures and Tables

**Table 1:** Primers used in this study.

Genes	Accession Number	Primer Sequences (5′–3′)	Product Size (bp)
***BMP-2***	XM_015283435.1	F: 5′-TCAGCTCAGGCCGTTGTTAG-3′ R: 5′-ACCCCACGTCATTGAAGTCC-3′	185
***RUNX-2***	AF_445419	F: 5′-TAAAGGTGACGGTGGATGG-3′ R: 5′-TGTGGATTAAAAGGACTTGGTG-3′	190
***GAPDH***	NM_204305.1	F: 5′-GCCCAGAACATCATCCCA-3′ R: 5′-CGGCAGGTCAGGTCAACA-3′	137

**Table 2:** Mortality rates.

Days	Control (n = 80)	TD (n = 80)	Osthole (n = 80)
1–7	2	9	7
8–10	2	5	2
11–14	1	1	0
15–18	0	1	1
Total dead birds	5	16	10

The mortality rate among control, TD, and osthole groups from day 1 to day 18. Chi-square analysis for total number dead and alive chickens.
